# Implementing the Eat, Sleep, Console (ESC) Model in a Small Safety-Net Hospital: A Phased Quality Improvement Initiative

**DOI:** 10.7759/cureus.110557

**Published:** 2026-06-09

**Authors:** Manali Ghadiali, Esther S Kim, Adam Freeman, Tristan Grogan, Rasika Venkateswaran, Supriya Bavisetty

**Affiliations:** 1 Pediatrics, Keck School of Medicine, University of Southern California, Los Angeles, USA; 2 Neonatology, Olive View/University of California Los Angeles Medical Center, Sylmar, USA; 3 Pediatrics, Olive View/University of California Los Angeles Medical Center, Sylmar, USA; 4 Medicine Statistics Core, David Geffen School of Medicine, University of California Los Angeles, Los Angeles, USA

**Keywords:** eat-sleep-console, finnegan scoring, implementation science, length of stay, maternal-infant bonding, neonatal opioid withdrawal syndrome, nicu utilization, non‑pharmacologic care, quality improvement, safety‑net hospital

## Abstract

Background and objective: The rising prevalence of opioid use during pregnancy has contributed to increasing rates of neonatal opioid withdrawal syndrome (NOWS). Traditional management using the Finnegan Neonatal Abstinence Scoring System (FNASS) is associated with prolonged hospitalization, increased neonatal intensive care unit (NICU) utilization, and reduced maternal‑infant bonding. Function‑based models such as Eat, Sleep, Console (ESC) emphasize non‑pharmacologic care and may improve outcomes. This project aimed to transition from FNASS to ESC within a small safety‑net hospital and evaluate associated clinical outcomes.

Methods: We conducted a single‑center quality improvement initiative using a phased implementation strategy over 2.5 years. Eligible neonates were >36 weeks gestation or >2000 grams with prenatal opioid exposure. Clinical outcomes were compared between infants managed with FNASS (n=24) and ESC (n=12). Due to the small sample size and skewed distributions, continuous variables were analyzed using Mann-Whitney U tests and reported as medians with interquartile ranges.

Results: ESC implementation was associated with shorter median length of stay (3.5 vs. 16.5 days; p<0.001), fewer days on pharmacologic treatment (0 vs. 11.5 days; p=0.008), and fewer days in the NICU (0 vs. 13.5 days; p<0.001). Infants in the ESC group spent a higher percentage of hospitalization bonding with their mothers (100% vs. 14.6%; p=0.002), although the total number of bonding days did not differ significantly (p=0.199). Pharmacologic treatment use was lower in the ESC group (0% vs. 54.2%; p=0.002).

Conclusions: Transitioning to ESC was associated with reduced pharmacologic treatment, shorter hospitalization, decreased NICU utilization, and improved maternal‑infant bonding. These findings support the feasibility of ESC implementation in resource‑limited safety‑net settings and highlight a scalable framework for equitable NOWS care.

## Introduction

Opioid use during pregnancy has increased substantially in the United States over the past decade, contributing to a parallel increase in neonatal opioid withdrawal syndrome (NOWS). Between 2010 and 2017, maternal opioid‑related diagnoses increased by 130%, while NOWS diagnoses rose by 82% across the United States [1]. In California, NOWS rates continue to rise, yet treatment protocols vary widely across hospitals [2]. NOWS results from in utero opioid exposure and can manifest postnatally with a spectrum of neurological, autonomic, gastrointestinal, and musculoskeletal symptoms [3]. Historically, management has relied on the Finnegan Neonatal Abstinence Scoring System (FNASS), a symptom‑based tool that evaluates 21 clinical signs of withdrawal [4]. A score of ≥12 once or ≥8 twice typically prompts pharmacologic intervention [5,6]. Despite widespread use, FNASS has demonstrated poor inter‑rater reliability and lacks validated score thresholds [7]. Emerging evidence suggests that FNASS‑guided care may prolong hospitalization, increase pharmacologic treatment, disrupt maternal‑infant bonding, and elevate healthcare costs [8-10].

To address these limitations, newer function‑based care models have been developed that emphasize non‑pharmacologic interventions and assess an infant's ability to eat effectively, sleep adequately, and be consoled when distressed [10-12]. Adoption of these approaches has been associated with reduced neonatal intensive care unit (NICU) utilization, shorter hospital stays, and enhanced parental involvement [12-14]. The financial impact of NOWS remains substantial, with estimated annual costs exceeding $1.5 billion [15,16].

Significant variability also existed in the use of pharmacologic versus non‑pharmacologic strategies [2]. A statewide survey found that although most institutions had at least one written guideline for NOWS management, implementation of newer function‑based models was inconsistent [2]. Given the challenges of standardizing care across diverse and resource‑limited settings, we aimed to transition from FNASS to a function‑based model at a safety‑net hospital in a stepwise and replicable manner. Our objective was to reduce pharmacologic treatment, minimize NICU exposure, enhance maternal‑infant bonding, and establish a scalable framework for equitable NOWS management.

## Materials and methods

This project was designed as a single‑center quality improvement initiative with a retrospective pre‑post evaluation of clinical outcomes. It was conducted at Olive View/University of California Los Angeles Medical Center, a safety‑net hospital in Los Angeles County that serves a diverse and medically underserved population. The hospital includes Maternal‑Child Health services and a level III NICU and averages 800-1,000 deliveries per year, with approximately 10-12 infants diagnosed with NOWS annually. The initiative aimed to improve care for neonates with NOWS by transitioning from FNASS to the Eat, Sleep, Console (ESC) model. Eligible neonates were those born at ≥36 weeks gestation or weighing ≥2000 grams with suspected or confirmed in utero opioid exposure, while infants requiring NICU‑level care for comorbidities were excluded. High‑risk mothers were identified through close collaboration with the obstetrics department.

**Figure 1:**
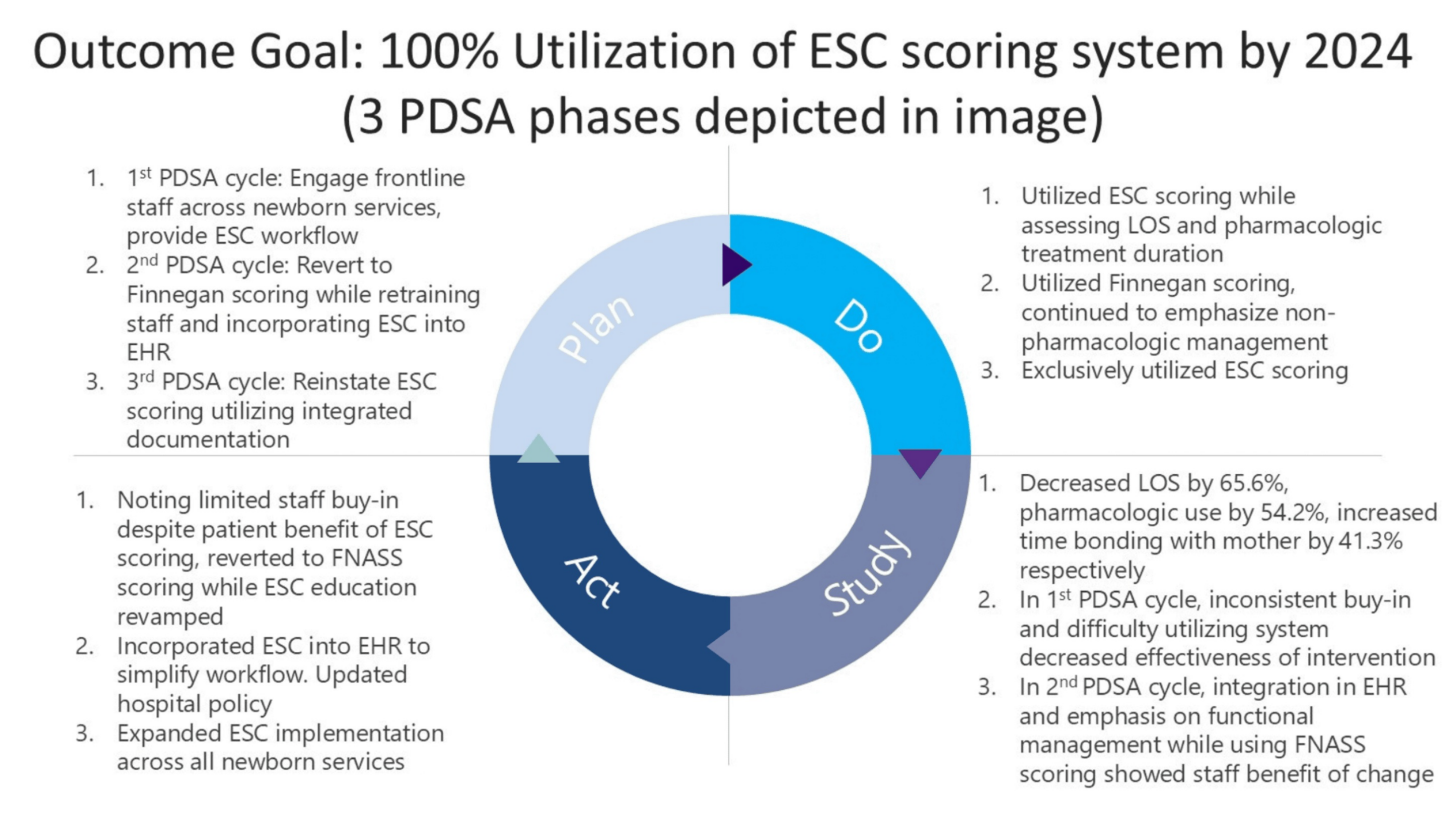
PDSA cycles used to implement the ESC workflow ESC: Eat, Sleep, Console; PDSA: Plan‑Do‑Study‑Act; EHR: electronic health record; FNASS: Finnegan Neonatal Abstinence Scoring System; LOS: length of stay The PDSA framework outlines the general planning, implementation, evaluation, and refinement steps undertaken to introduce the ESC scoring system and how the intervention was re-evaluated from a workflow perspective to ensure long-term utilization.

The intervention was implemented over 2.5 years using a phased approach grounded in the Plan‑Do‑Study‑Act (PDSA) framework, which guided iterative planning, implementation, evaluation, and refinement of the ESC model (Figure 1). Phase 1 (June 2022-July 2023) introduced ESC scoring in the newborn nursery with lateral transfer to the pediatric floor, emphasizing non‑pharmacologic management. However, inconsistent training and limited staff buy‑in prompted a temporary return to FNASS scoring during Phase 2 (August 2023-October 2024), although non‑pharmacologic care continued. During this period, standardized ESC training was delivered, the protocol was formalized into hospital policy, and electronic health record (EHR) tools were developed in collaboration with the information technology department. Phase 3 (October 2024-December 2024) reinstated ESC scoring hospital‑wide, supported by trained staff and integrated documentation.

Primary outcome measures included average hospital length of stay, days of pharmacologic treatment, days of hospitalization spent in the NICU, and the percentage of hospitalization spent bonding with the mother, quantified by rooming‑in time. Process measures included staff training completion rates and EHR documentation compliance. Balancing measures included monitoring for adverse events such as readmission for withdrawal symptoms within two weeks of discharge. Within the ESC scoring system, feeding is included; therefore, infants with elevated ESC feeding scores received an occupational therapy consultation for a formal feeding evaluation prior to discharge. An additional balancing measure was readmission for poor weight gain within two weeks of discharge. Poor weight gain was attributed to withdrawal only if the infant presented with an elevated ESC score and after the exclusion of other common causes of neonatal weight loss, such as inadequate intake, breastfeeding challenges, or dehydration.

Clinical outcomes were compared between neonates managed with FNASS (n=24) and those managed with ESC (n=12). Data were collected retrospectively from medical records, and pre‑implementation data were obtained through chart review covering January 2020-May 2022. Patient characteristics and outcomes were summarized across the 2.5‑year implementation period as well as by management group. Continuous variables were summarized using means and standard deviations or medians and interquartile ranges, as appropriate based on distribution. Given the small sample size and skewed distributions for key outcome variables, between-group comparisons for these outcomes were performed using Mann-Whitney U tests. Categorical variables were summarized using frequencies and percentages and compared using Fisher's exact tests due to small cell counts. Apgar scores were treated as ordinal/categorical variables and compared using Fisher's exact tests. Between‑group comparisons used two‑sample t‑tests for continuous variables. Statistical significance was defined as p<0.05.

To illustrate temporal changes in practice and outcomes, we constructed a time-series plot of average length of stay and average days on pharmacologic treatment across the implementation period, stratified by major phases of program adoption (pre-ESC, ESC introduction, transition period with mixed scoring, and live EHR integration). This descriptive visualization was used to highlight trends over time, recognizing the phased and somewhat overlapping implementation of ESC. All analyses were conducted using R Version 4.4.3 (R Foundation for Statistical Computing, Vienna, Austria).

Ethical considerations

Approval was obtained from the Institutional Review Board of the Olive View/University of California Los Angeles (UCLA) Education and Research Institute (approval number: 2266328-3) before study commencement. Patient confidentiality and data security were maintained throughout all phases of implementation. In alignment with the Standards for QUality Improvement Reporting Excellence (SQUIRE) 2.0 reporting guidelines, this manuscript describes the clinical context, rationale for the intervention, phased implementation strategy, study of the intervention using outcome, process, and balancing measures, and analytical methods used to evaluate change over time. The Results and Discussion sections address contextual factors, unintended consequences, interpretation of findings, and limitations consistent with SQUIRE 2.0 expectations.

## Results

**Figure 2:**
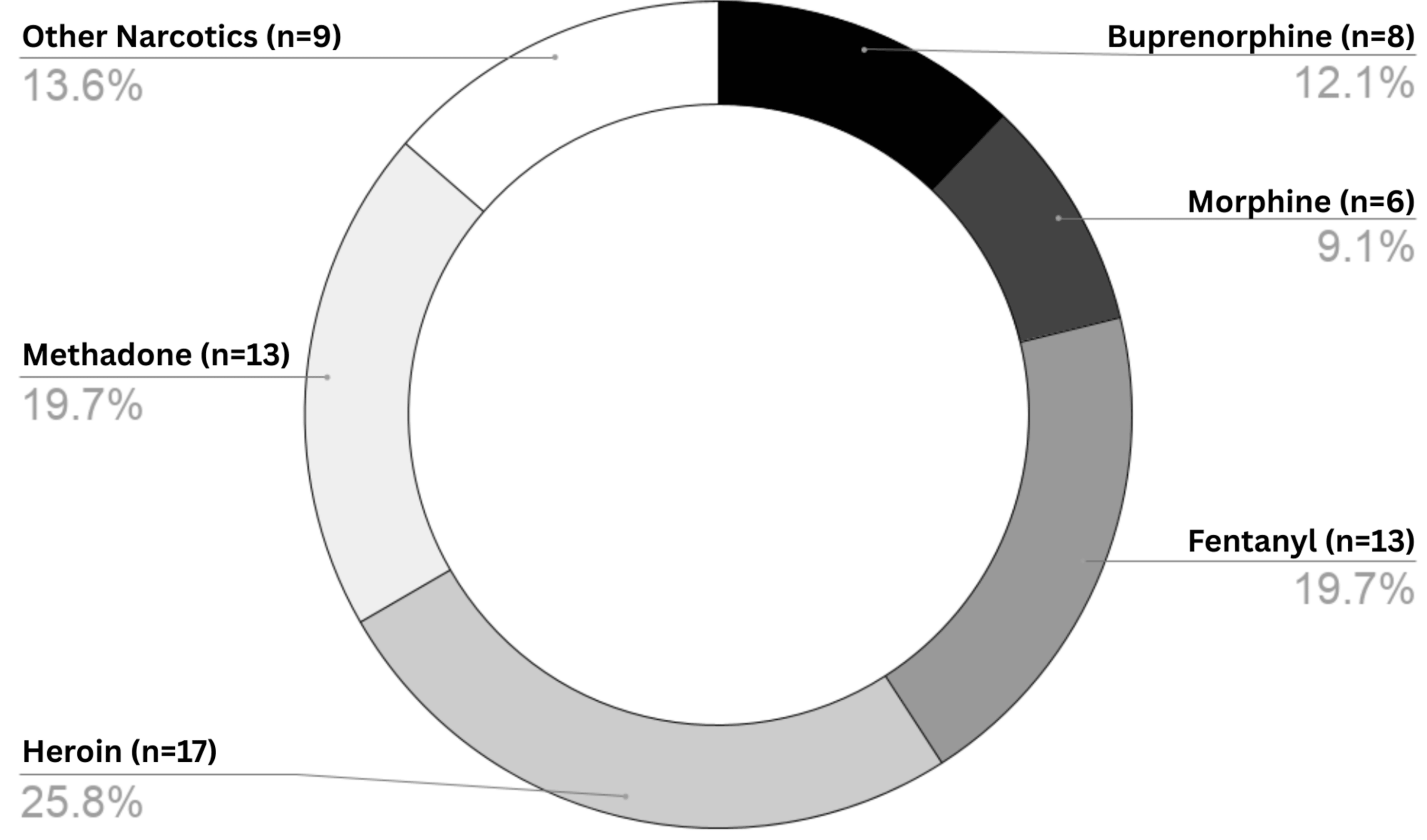
Distribution of opioid exposures among infants with prenatal opioid exposure Donut chart demonstrating the distribution of specific opioid types identified in the cohort: heroin (25.8%), methadone (19.7%), fentanyl (19.7%), other narcotics (13.6%), buprenorphine (12.1%), and morphine (9.1%). Infants with polysubstance exposure are represented across multiple categories; therefore, the total number of exposure entries is greater than the number of unique infants in the cohort (n=36).

This project focused only on opioid exposure. All infants in the cohort had polysubstance exposure, and many had more than one opioid exposure during pregnancy. Opioid exposures included fentanyl, heroin, methadone, buprenorphine, morphine, and other narcotics. Exposure profiles are illustrated in Figure 2.

Table 1 summarizes patient characteristics across the 2.5‑year implementation period and by management group (ESC vs. FNASS). No statistically significant differences were observed between groups in baseline demographic or perinatal characteristics.

**Table 1:** Patient demographics ESC: Eat, Sleep, Console; FNASS: Finnegan Neonatal Abstinence Scoring System; C-section: cesarean section; NSVD: normal spontaneous vaginal delivery Data are presented as mean (standard deviation) or n (percentage). Continuous variables were compared using two‑sample t‑tests and are reported with corresponding t statistics and degrees of freedom. Categorical variables were compared using Fisher's exact tests due to small cell counts. A p‑value of <0.05 was considered statistically significant. Small for gestational age was defined as birth weight below the 10th percentile.

Variable	Category	Total	ESC (n=12)	FNASS (n=24)	Test statistic	P-value
Gender	Female	15 (41.7%)	5 (41.7%)	10 (41.7%)	N/A: Fisher's exact test	1
	Male	21 (58.3%)	7 (58.3%)	14 (58.3%)		
Delivery mode	C-section	13 (36.1%)	6 (50%)	7 (29.2%)	N/A: Fisher's exact test	0.281
	NSVD	23 (63.9%)	6 (50%)	17 (70.8%)		
Small for gestational age	-	4 (11.1%)	2 (16.7%)	2 (8.3%)	N/A: Fisher's exact test	0.588
Gravida	-	3.8 (2.7)	3.7 (2.2)	3.8 (3.0)	t(34)=-0.127	0.9
Para	-	2.3 (1.3)	2.5 (1.3)	2.2 (1.3)	t(34)=0.544	0.59
Apgar score at 1 min	3	1 (2.8%)	1 (8.3%)	0 (0%)	N/A: Fisher's exact test	0.327
	5	1 (2.8%)	1 (8.3%)	0 (0%)		
	6	2 (5.6%)	1 (8.3%)	1 (4.2%)		
	7	3 (8.3%)	0 (0%)	3 (12.5%)		
	8	25 (69.4%)	8 (66.7%)	17 (70.8%)		
	9	4 (11.1%)	1 (8.3%)	3 (12.5%)		
Apgar score at 5 min	7	2 (5.6%)	1 (8.3%)	1 (4.2%)	N/A: Fisher's exact test	1
	8	5 (13.9%)	1 (8.3%)	4 (16.7%)		
	9	29 (80.6%)	10 (83.3%)	19 (79.2%)		
Multiple gestation	-	0 (0%)	0 (0%)	0 (0%)	N/A: Fisher's exact test	1

Implementation of the ESC model was associated with significant improvements in several clinical outcomes. Compared with infants managed using FNASS, those in the ESC group had a markedly shorter median length of stay (3.5 vs. 16.5 days; p<0.001). ESC‑managed infants also required fewer days of pharmacologic treatment (0 vs. 11.5 days; p=0.008) and spent fewer days in the NICU (0 vs. 13.5 days; p<0.001). The percentage of hospitalization spent bonding with the mother was substantially higher in the ESC group (100% vs. 14.6%; p=0.002), although the total number of bonding days did not differ significantly between groups (p=0.199). Pharmacologic treatment use was significantly lower among ESC‑managed infants (0% vs. 54.2%; p=0.002). These findings reflect consistent improvements across multiple domains of care following ESC implementation and align with the function‑based model's emphasis on non‑pharmacologic management and parental involvement (Table 2).

**Table 2:** Clinical outcomes by management group using median (IQR) and non‑parametric testing ESC: Eat, Sleep, Console; FNASS: Finnegan Neonatal Abstinence Scoring System; IQR: interquartile range; NICU: neonatal intensive care unit Median values with IQR for key clinical outcomes comparing infants managed with the ESC versus the FNASS scoring system. Due to skewed distributions and small sample sizes, between‑group comparisons were conducted using the Mann-Whitney U test for continuous variables. A p‑value of <0.05 was considered statistically significant.

Variable	Total (N=36)	ESC (N=12)	FNASS (N=24)	Test	P-value
Length of stay	7.5 (3.8, 21.0)	3.5 (2.8, 5.0)	16.5 (6.5, 23.0)	Mann-Whitney U test	<0.001
Days on pharmacologic treatment	0 (0.0, 15.5)	0 (0.0, 0.0)	11.5 (0.0, 20.5)	Mann-Whitney U test	0.008
Days bonding with mom	2 (0.0, 4.0)	3 (2.0, 4.2)	1 (0.0, 4.0)	Mann-Whitney U test	0.199
% of hospital bonding days with mom	63.3 (0.0, 100.0)	100 (100.0, 100.0)	14.6 (0.0, 70.2)	Mann-Whitney U test	0.002
Days in the NICU	1 (0.0, 20.0)	0 (0.0, 0.0)	13.5 (0.0, 21.5)	Mann-Whitney U test	<0.001
% of hospital in the NICU	9.5 (0.0, 100.0)	0 (0.0, 0.0)	85.4 (0.0, 100.0)	Mann-Whitney U test	0.003
Pharmacologic treatment used	13 (36.1%)	0 (0%)	13 (54.2%)	Fisher's exact test	0.002

**Figure 3:**
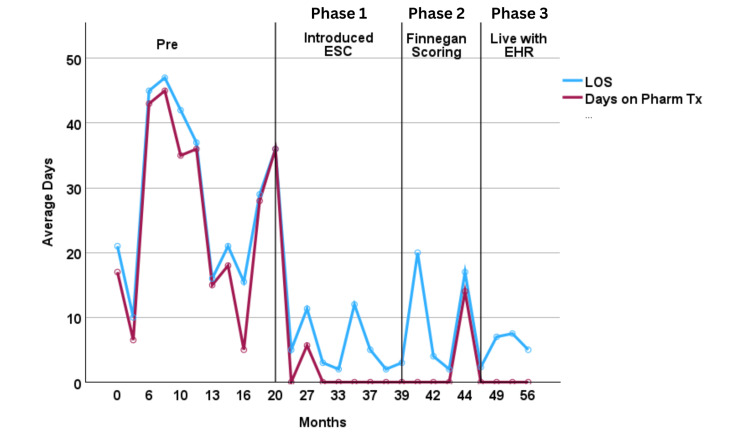
Trends in LOS and pharmacologic treatment across implementation phases ESC: Eat, Sleep, Console; FNASS: Finnegan Neonatal Abstinence Scoring System; EHR: electronic health record; Tx: treatment; LOS: length of stay; NOWS: neonatal opioid withdrawal syndrome Temporal trends in average hospital LOS and days of pharmacologic treatment for infants with NOWS across the pre‑implementation period and three ESC implementation phases, demonstrating higher values during FNASS‑dominant periods and substantial reductions following ESC adoption.

Figure 3 shows the temporal trends in average hospital length of stay and days of pharmacologic treatment for infants with NOWS across the pre‑implementation period and the three ESC implementation phases. Length of stay and medication use remained high during FNASS‑dominant periods and declined substantially following ESC adoption. Phase labels (ESC introduction, FNASS reinstatement, ESC with EHR integration) highlight the relationship between implementation milestones and clinical outcomes. This figure is intended for descriptive purposes only; no interrupted time‑series analysis was performed.

No adverse events occurred during the ESC implementation period. No infants required readmission for withdrawal symptoms within two weeks of discharge. Occasional delays in discharge were related to Department of Child and Family Services (DCFS) placement decisions rather than clinical instability. These findings support the safety, feasibility, and clinical effectiveness of ESC implementation in a resource‑limited setting.

## Discussion

This quality improvement initiative sought to improve the management of NOWS in a safety‑net hospital by transitioning from the symptom‑based FNASS approach to the function‑based ESC model. The phased implementation strategy resulted in substantial reductions in pharmacologic treatment, NICU utilization, and overall hospital length of stay while simultaneously increasing maternal‑infant bonding time. These findings align with emerging evidence demonstrating the effectiveness of ESC‑based care in reducing unnecessary interventions and promoting family‑centered practices.

The rising incidence of NOWS, combined with prolonged hospitalizations, contributes significantly to healthcare costs in the United States [15,16]. Our results demonstrate that ESC can be successfully implemented in a resource‑limited safety‑net setting without additional operating or capital expenditures. By caring for medically stable infants in postpartum or pediatric units rather than the NICU, the hospital reduced NICU admissions and improved opportunities for rooming‑in and parental engagement. Collaboration among physicians, nursing leadership, and informatics teams facilitated the integration of ESC scoring into the EHR, ultimately supporting a hospital‑wide policy change.

Phase 1 of implementation revealed gaps in infrastructure, including inconsistent staff training and the absence of EHR‑integrated tools. These challenges contributed to variable adoption and prompted a temporary return to FNASS scoring during Phase 2. Despite this reversion, the continued emphasis on non‑pharmacologic care led to meaningful reductions in medication use, suggesting that improvements were attributable not solely to scoring methodology but also to strengthened care practices. During Phase 2, the team standardized ESC training, formalized the protocol, and developed EHR documentation tools, which collectively enabled successful hospital‑wide implementation in Phase 3.

**Figure 4:**
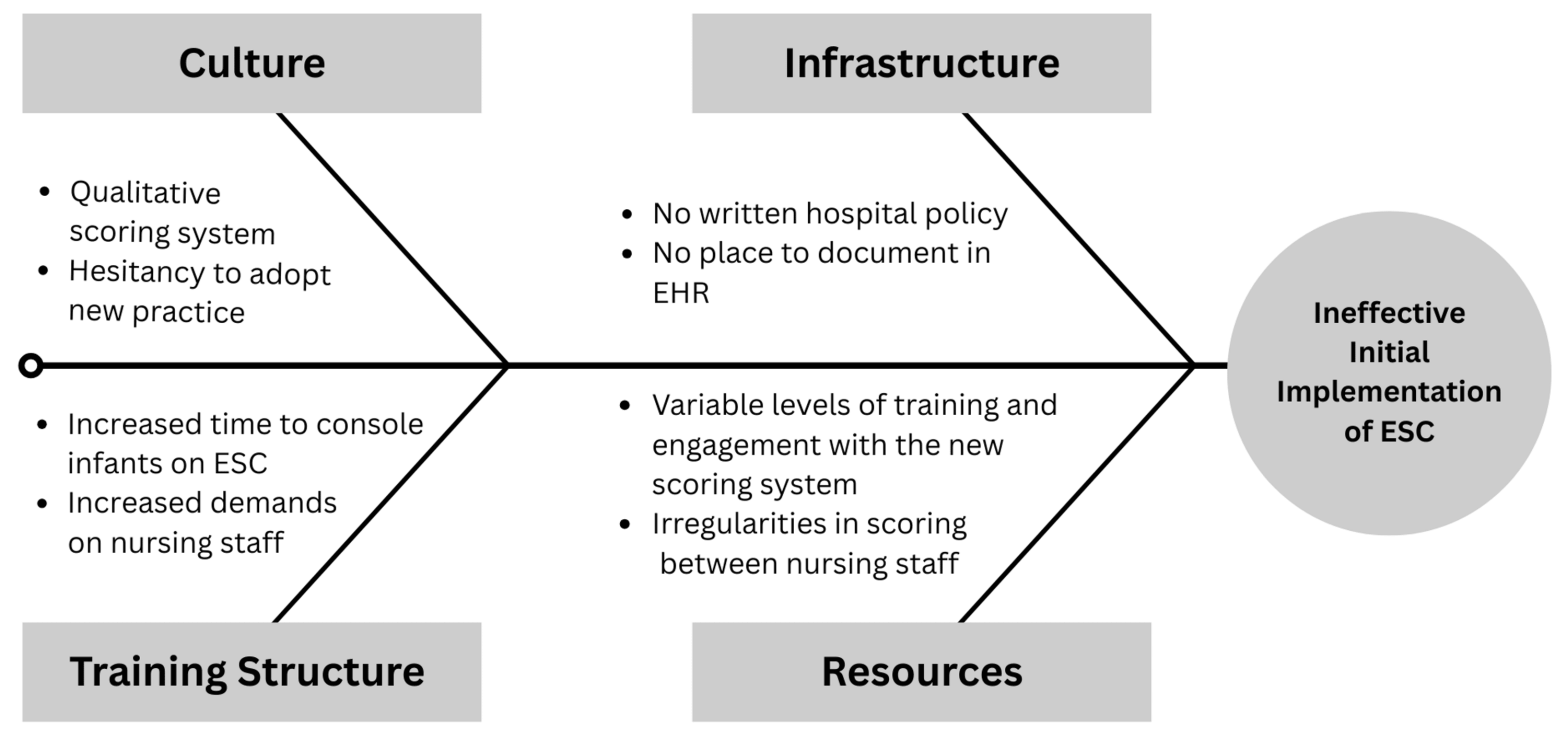
Fishbone diagram illustrating the four key categories of culture, infrastructure, training structure, and resources and how each contributed to the ineffective initial implementation of the ESC model ESC: Eat, Sleep, Console; PDSA: Plan‑Do‑Study‑Act; EHR: electronic health record Diagram created using Canva (Canva Pty Ltd., Sydney, Australia)

The transition from symptom‑based to function‑based assessment required a significant cultural shift. Staff hesitancy was addressed through repeated educational sessions, interdisciplinary meetings, and sharing of successful outcomes from other institutions. These efforts fostered broader acceptance of ESC and improved consistency in its application. A fishbone analysis identified barriers related to culture, infrastructure, training, and resources, guiding targeted improvements (Figure 4).

Determining the appropriate monitoring period was complicated by variability in withdrawal onset based on opioid type. Although national guidelines recommend 96-120 hours of observation for infants exposed to long‑acting opioids [7], our institution used a minimum 48‑hour monitoring period within the ESC framework. Heroin was the most common opioid exposure in our cohort, followed by methadone, fentanyl, buprenorphine, morphine, and other narcotics. Because all infants had polysubstance exposure and many had more than one opioid exposure, the team selected a 48‑hour minimum observation period tailored to the pharmacokinetics of the most frequently encountered, shorter‑acting opioids. For substances such as heroin and fentanyl, withdrawal symptoms typically emerge within the first 48 hours if they are going to escalate. During this observation period, clinicians assessed both infant functional stability and the family's ability to provide consistent non‑pharmacologic care. If there was any concern for escalating withdrawal symptoms at the 48‑hour mark, physicians were encouraged to use their clinical discretion to continue inpatient observation rather than discharge. All infants had a follow‑up visit scheduled within 48 hours of discharge, aligned with the end of the monitoring window recommended in national guidelines. No infants required readmission for withdrawal within two weeks of discharge, supporting the safety of this shortened observation period when paired with ESC‑based assessment and robust follow‑up.

Non‑pharmacologic care increased nursing workload due to the need for prolonged soothing and holding. To address this, the team identified additional support persons, including volunteers and care partners, to assist with infant care, helping distribute workload and maintain care quality. Mothers reported feeling more connected to their infants compared with prior births involving NICU admission, and bonding time increased by 59%. Early identification of support persons also facilitated smoother transitions to foster care when necessary. Nursing staff found ESC scoring simpler and more intuitive than FNASS, improving workflow and care delivery.

This study has several limitations. The sample size, particularly within the ESC cohort, was small and may limit the precision and generalizability of findings. As a single‑site quality improvement initiative in a safety‑net hospital, results may not fully reflect practices or resources in other settings. Although most infants in our population are typically followed within the same health system network due to insurance coverage, there remains a small possibility that a readmission at an outside facility could have been missed. Additionally, because ESC implementation included a bundle of non‑pharmacologic interventions, it is difficult to isolate the independent effect of ESC scoring from additional nursing comfort following training as well as the broader care bundle, introducing the potential for confounding. Finally, while ESC emphasizes non‑pharmacologic care, there remains a potential risk of under‑treatment if clinicians perceive pharmacologic therapy as inconsistent with the model. These limitations highlight the need for larger, multi‑site evaluations to confirm the durability, safety, and generalizability of ESC implementation across diverse populations.

Future directions include expanding our efforts through the Plan of Safe Care (POSC) initiative, which provides longitudinal support for pregnant and postpartum mothers with substance use. Supported by DCFS and funded through a grant awarded to the Office of Child Protection (OCP), our institution is now a pilot site in which a POSC navigator will be assigned to provide ongoing outreach, education, and linkage to services for families identified antenatally or at delivery. This program addresses the needs of a highly vulnerable population who often face the risk of separation due to DCFS involvement and may avoid seeking care out of fear of custody loss. By proactively supporting maternal sobriety, strengthening family stability, and connecting families with community resources, the initiative aims to reduce neonatal opioid exposure, prevent unnecessary separations, and improve long‑term outcomes for both mothers and infants.

## Conclusions

ESC implementation was associated with reductions in pharmacologic treatment, length of stay, and NICU utilization. These findings suggest that function‑based care may support safer, less intervention‑intensive management of infants with prenatal opioid exposure. Reduced NICU use and shorter hospital stays may potentially decrease hospital costs, although a formal cost analysis was not performed. Importantly, the intervention leveraged existing staff and resources, underscoring its feasibility in resource‑limited settings.

Overall, a phased implementation strategy with repeated provider and nursing education, early engagement of informatics and hospital leadership, and incorporation of patient support persons appears to facilitate successful adoption of the ESC model. This structured approach may help other institutions transition from symptom‑based to function‑based care for infants at risk of neonatal opioid withdrawal.
